# Rochester’s Outdoor School Where Delicate Children Get Well and Strong

**Published:** 1911-06-15

**Authors:** A. M. Dickinson

**Affiliations:** Managing Editor, Utica Globe


### ROCHESTER'S OUTDOOR SCHOOL WHERE DELICATE CHILDREN GET WELL AND STRONG.

A. M. DICKINSON, MANAGING EDITOR, UTICA GLOBE.

"And not one of them has had a cold this winter."

The speaker was Miss Catherine Fichtner, principle of Rochester's outdoor school on Scio Street, and there was a ring of triumph in her voice. The day was one of the worst of the entire winter. The mercury was but few degrees above zero and the wind was blowing a gale, sweeping around corners, searching out every nook and cranny with its icy breath and hurling the frozen snow with almost the force and cutting effect of a sand blast.

Dressed as ordinarily for the street, with a medium weight overcoat and heavy suit, I was chilled to the bone and shivering from head to foot as I tried to take notes with my gloved fingers. On the other hand Miss Fichtner and her assistant, Miss Jessie Sickles, and all the boys and girls in their charge were as warm and as comfortable as a farmer's boy on the sunny side of a barn in May. Why the difference? Well, I was just a newspaper man garmented for ordinary outdoor duty, while the two ladies were the teachers and the children were the pupils in Rochester's outdoor school and they were dressed for their parts. No Eskimo in his polar bear suit and walrus skin boots was ever more absolutely defiant of winter's keenest rigors than were these two ladies and the rosy youngsters in their care. It made no difference to them that the wind raged and that the frost was driving to cover everyone whom stern necessity did not compel to face the elements.

And these children? No casual observer would have guessed that they were sick—too weak and anaemic to attend the ordinary public schools. They didn't look like a bunch of youngsters whom the doctors had selected as threatened with consumption or general decline. They were rosy of face, plump and lively, and they did look like a lot of minature Artic explorers. Each boy was dressed in a heavy blanket coat and trousers and each girl wore a heavy blanket skirt. Over each head was drawn a blanket hood such as the French Canadian woodsmen call a "capote,"and each pair of feet and legs was encased in heavy lumberman's felt boots. Hands were protected either with mittens or heavy gloves. So garbed and absolutely defiant of weather conditions these happy youngsters study, recite, eat, sleep and play—and, better than all else, they daily grow stronger and fatter and more able to battle against the weak constitutions and tendency to disease with which either inheritance or environment had encumbered them.

It's an interesting story, that of the outdoor school, and as we watched the children at work or at play Miss Fichtner told me something about it.

### ORIGINATED IN GERMANY.

The outdoor school originated in Germany, a country which for many years has stood in the front ranks in matters educational. In 1904 a school for delicate and convalescing children was established in a pine woods of four acres in

Charlottenburg, a suburb of Berlin, and within a couple of years the school had 250 pupils and 9 teachers, a trained nurse and several helpers. All expenses were paid by the municipality and the experiment was proved to be a grand success.

Providence, R. I., was the first American city to adopt the idea, though hers is a fresh air rather than an outdoor school. Other cities to swing into line, each with entire success, are Boston, Chicago, Pittsburg, Buffalo, New York City, Montclair, N. J., and Rochester. In Boston and Montclair the children's limbs and lower part of their bodies are encased in bags, but the other cities adopt the blanket trousers and skirts for the reason that the latter permit of greater activity on the part of the children.

In Rochester the children who are to have the benefit of the outdoor school are selected from the common schools on the report of the principle or visiting nurse. Found to be below the normal in health the children are examined, if found weak and anaemic and at the same time not mentally deficient or incorrigible or so far advanced in disease as to be a menace to others, they are assigned to the outdoor school. They are selected from all grades from the first to the eighth inclusive. On arrival they are carefully examined and a record is made of their weight, pulse, temperature and general condition. This examination is repeated at frequent intervals and their progress toward recovery is kept in tabulated form easy of comprehension. When they reach a normal condition they are returned to their regular classes to make room for others.

The department of education equips the school and the dining-room and furnishes the sleeping chairs. The Rochester Public Health Association, an orginazation supported by private subscription supplies the suits, the food, the combs, the tooth brushes and cups, and pays the wages of the cook. Some of the children come long distances and for such of them as is necessary street car fare is provided. As they get stronger—and they all do get stronger—they are encourged to walk back and forth, unless the distance is great. In the latter case they are urged to walk at least part of the way.

FURNISHED WITH PLENTY TO EAT.

On arrival at the school in the morning the children are furnished with a dish of oatmeal and milk. At 10:50 they are given a glass of milk, which they drink out of doors unless the weather is too severe. At 12 o'clock dinner is served in the dining-room. The dinner consists of meat, potatoes, bread and butter and milk, one or more vegetables and dessert. The greatest attention is paid to food values in making up the menue. At 3:30 each child is given another glass of milk or a cup of hot cocoa or a dish of cream soup with crackers. At each of these meals or lunches boys and girls are detailed to serve and afterwards to wash the dishes. It is a duty which they all like and all eagerly keep track of the days when they are to officiate. At first only the girls were detailed to wash dishes, but the boys got up a petition and asked to have the privilege extended to them. The sense of responsibility is very strong and each detail tries to make a record for perfect service. Nothing in the way of neatness or attention is neglected. I was present when milk was served and I absentmindedly sipped the nourishing liquid as I talked with Miss Fitchtner. I had not noticed that my glass was empty until I glanced up to see a neat little chap standing in front of me holding out a tray for the glass. He had not been prompted, but, as the poet has said, "he seen his duty and he done it." At the meals as at all other times during the school sessions the older and stronger children treat the smaller and weaker with every consideration and seem to be imbued with a spirit of mutual help.

The children are taught the virtue of personal neatness and three times a day each little set of teeth is carefully cleaned. And, by the way, Rochester pays great attention to the care of the teeth of her school children, not only those attending the outdoor school but those in all the ward schools. The teeth of every child are examined at regular intervals and at the appearance of the slightest trouble the children are sent to the dental clinic maintained in two of the schools by the Rochester Dental Society.

### SCHOOL IN A TENT.

Rochester's outdoor school was begun October 4, 1909, in a tent in the country, but a great storm tore down the tent and carried it away. Then the school was moved to open rooms at School No. 2 and finally to its present location on Scio street, where it is conducted in a large tent. In pleasant weather all sides of this tent are open, but during periods of extreme cold or wind the tent is partially closed. At least one side, however, is always open. The tent is divided into two compartments. One is used as a school room proper, with desks, seats and blackboards. Here the children study and recite, receiving from the teachers that individual attention which they are deprived of in great measure in the regular schools. The other compartment is furnished with steamer chairs and blankets. Here for an hour and a quarter each day after the main meal, the children lie and rest. One of the teachers reads aloud to them for a few minutes, at the end of which time most of them are sound asleep. During the rest period no play or talking is permitted. At the end of the time set for the rest the children are turned out for a brief romp to get their blood in circulation before resuming their work. No child is deliberately wakened, however, and some little ones, especially when they first come to the school, sleep until time to go home at 4 o'clock.

Eat, play, study, recite, exercise, eat, sleep, play, study, exercise, etc., etc., etc., This is the program throughout the long day, which the children find all too short to suit them. They love the school and would be there Saturdays and Sundays if permitted. They consider the birthdays of Washington and Lincoln a hardship, for they mean no school. The saddest words which can come to their ears are those uttered by the nurse or the visiting physician when she or he pronounces them normal and fit to return to the regular schools. One little girl when she heard the dreaded words began to weep piteously and to lament, "I don't want to be normal! I don't want to be normal!"

Last year the school was conducted during the entire summer and that plan will be followed this year, with a couple of week's visit to the country.

This summer a garden will be conducted. Already a hotbed has been made and just as soon as the weather and the ground are right for it the children will spend their play periods in setting out and caring for a variety of vegetables and flowers. The former will be used in the kitchen and the later for decorative purposes, thus teaching the pupils valuable lessons. From the beginning the children have had pots and bulbs and they watch the development with much interest taking the greatest pride in being able to produce flowers for the dining-room or to carry home.

The dining-room, by the way, is the only indoor feature of the school. It is in the main school building and is kept comfortably warm. The main meal is always eaten here and the children are encouraged to go to this room and get warm if at any time they feel the need of it. But a constant effort is made to keep them comfortable by means of warm clothing and exercise.

### MENTAL AND PHYSICAL GAIN.

One might think that with such a school and with so much time devoted to exercise, eating and sleeping, the children would fall behind in their classes. But such is not the case. When restored to health they go back to their regular classes and are able to keep up with the work. In one instance a girl, when she returned to the ward school, was found so far advanced that she was permitted to enter a higher grade, skipping a whole class.

It would be impossible to exaggerate the beneficial influence which the school has on the health of the children. They come pale and anaemic, weak and listless. They go away ruddy, strong and full of energy. In three months the average gain in weight was nearly 6 pounds. One girl gained 10½ pounds in this time. That this improvement is due entirely to the school treatment is proved by the fact that during the recent Christmas holidays when for 13 days the children were at home, out of 17 of whom a record was kept 6 gained an average of 14 ounces and 10 lost an average of a pound and six ounces.—*The Dispensary Record.*

